# Progress towards understanding risk factor mechanisms in the development of autism spectrum disorders

**DOI:** 10.1042/BST20231004

**Published:** 2024-09-02

**Authors:** Amelia Bryers, Cheryl A. Hawkes, Edward Parkin, Neil Dawson

**Affiliations:** Division of Biomedical and Life Sciences, Faculty of Health and Medicine, Lancaster University, Lancaster, UK.

**Keywords:** GABA, glutamate, mouse models, neurodevelopmental disorders, neuroimaging, serotonin

## Abstract

Autism spectrum disorders (ASD) are a heterogenous set of syndromes characterised by social impairment and cognitive symptoms. Currently, there are limited treatment options available to help people with ASD manage their symptoms. Understanding the biological mechanisms that result in ASD diagnosis and symptomatology is an essential step in developing new interventional strategies. Human genetic studies have identified common gene variants of small effect and rare risk genes and copy number variants (CNVs) that substantially increase the risk of developing ASD. Reverse translational studies using rodent models based on these genetic variants provide new insight into the biological basis of ASD. Here we review recent findings from three ASD associated CNV mouse models (16p11.2, 2p16.3 and 22q11.2 deletion) that show behavioural and cognitive phenotypes relevant to ASD. These models have identified disturbed excitation-inhibition neurotransmitter balance, evidenced by dysfunctional glutamate and GABA signalling, as a key aetiological mechanism. These models also provide emerging evidence for serotoninergic neurotransmitter system dysfunction, although more work is needed to clarify the nature of this. At the brain network level, prefrontal cortex (PFC) dysfunctional connectivity is also evident across these models, supporting disturbed PFC function as a key nexus in ASD aetiology. Overall, published data highlight the utility and valuable insight gained into ASD aetiology from preclinical CNV mouse models. These have identified key aetiological mechanisms that represent putative novel therapeutic targets for the treatment of ASD symptoms, making them useful translational models for future drug discovery, development and validation.

## Introduction

Autism spectrum disorders (ASD) are a heterogenous set of syndromes characterised by social impairments and communication deficits, often accompanied by restrictive, repetitive behaviours and interests and altered sensory processing [1]. The prevalence of ASD diagnosis has increased over recent decades. In 2020 prevalence was estimated at ∼1 in 34 children in the U.S.A., an increase from 1 in 68 in 2010, with diagnosis being four times more common in males than females [2]. Given the complexity and multifactorial nature of ASD there is no singular disease mechanism that underlies diagnosis. However, research has identified key aetiological mechanisms that contribute to the risk of developing ASD, highlighting some of the central biological mechanisms involved. This review summarises several key ASD risk factor mechanisms, with a focus on recent insights gained from rodent models based on genetic risk for ASD and the impact on neurotransmitter system function.

## The genetic risk basis of ASD

Heritability estimates for ASD range from 40% to 90% [3,4], emphasising the central importance of genetic risk. Environmental risk factors, such as maternal smoking, advanced paternal age and prenatal maternal infection are also important [5–7], with at least some of these interacting with genetic risk [8]. Protective modifiable factors in relation to an individual's neurodevelopmental trajectory are also important, and diagnostic outcome is often dissociated from genetic risk. Such protective factors include a healthy maternal body weight (both pre- and during pregnancy), good maternal nutrition and breastfeeding [9–12].

The genetic architecture of ASD risk is highly complex and polygenic in nature. In most cases, ASD results from the interaction of multiple common genetic risk variants, with each variant individually contributing a very small increase in risk. Genome Wide Association Studies (GWAS) with very large sample sizes have been key in identifying common gene polymorphisms that contribute to an increased risk of developing ASD. These studies have provided important new insights into some of the key biological mechanisms contributing to ASD risk, including genetic mutations that impact neuronal gene transcription (*ASH2L*, *BCL11A*, *KANSL1*, *SOX7*, *SRRM4*, *XRN2*), brain development (*BLK*, *BTG1*, *CNTN5*, *DSCAM*, *FOXP1*, *KIZ*, *MMP2*, *NTM*, *WDR73*, *WNT3*, *XKR6*), inflammation (*C2CD4A*, *IFI16*, *MFHAS1*, *NFKB2*), the stress response (*CRHR1*), neuronal cell activity (*DPP10*, *KCNN2*) and synaptic (*CNTNAP5*, *DDHD2*, *SEMA3G*, *SNCA*) and neurotransmitter system (*GABBR2*, *GRIN2A*, *PAFAH1B1*, *RASGEF18B*) function [13–15].

In addition to common gene variants of small effect, single genes and copy number variants (CNVs), wherein chromosomal segments are either deleted or duplicated, that substantially increase the risk of developing ASD have also been identified (Table 1). CNVs have received particular interest following the observation that rare and *de novo* CNVs are more prevalent in ASD populations [16,17]. CNVs result in a gain or loss of genetic material that vary in size from tens of thousands to millions of nucleotides, altering the number of copies of a particular gene (e.g. 2p16.3 deletion, *NRXN1*) or multiple genes (e.g. 16p11.2 deletion/duplication, ∼29 genes), depending on the CNV. Importantly, some genes located in CNV regions associated with ASD have been independently identified as having common variants that are also associated with ASD diagnosis [14,18,19]. The Simon's Foundation Autism Research Initiative (SFARI) Gene database provides a valuable, extensive database that rationalises the contribution of risk genes and CNVs for ASD (https://gene.sfari.org).

**Table 1.** Single risk genes and CNVs that substantially increase the risk of ASD diagnosis.

Monogenic disorder/CNV syndrome	Gene(s) involved^a^	ASD odd's ratio (OR)/hazard ratio (HR)/% population	Key biological mechanisms and phenotypic outcomes
Fragile X	Fragile X messenger ribonucleoprotein 1 (*FMR1*)	-	Transcriptional regulation including the regulation synaptic mRNAs (Darnell et al., 2011, PMID: 21784246) [77]
Rett's syndrome	Methyl-CpG binding protein 2 (*MECP2*)	-	Regulation of neuronal transcriptional programmes (Tillotson and Bird, 2020, PMID: 31629770) [78]
2p16.3 deletion	Neurexin-1 (*NRXN1*)	∼14.9 OR (Matsunami et al., 2013, PMID: 23341896 [34]; Wang et al., 2017, PMID: 29045040 [35]; Yuen et al., 2017, PMID: 28263302 [79])	Synaptic protein which binds to post-synaptic partners to regulate synaptic maturation and function, including glutamate synapse function (Gomez et al., 2021, PMID: 33420412 [36]; Sudhof, 2017, PMID: 29100073 [37])
16p11.2 deletion/duplication	∼29 genes including Mitogen-activated protein kinase 3 (*MAPK3*), *Thousand and one amino-acid kinase 2 (TAOK2)*, *Major Vault Protein (MVP)*	Deletion = ∼38.7 OR Duplication = ∼20.7 OR (Walsh and Bracken, 2011, PMID: 21289514 [80])	*MAPK3*: Extracellular signal regulated kinases essential for cell proliferation, differentiation, and cell cycle progression (Boutros, Chevet & Metrakos, 2008, PMID: 18922965 [81]) *TAOK2*: Activates the p38 kinase cascades through activation of MEK kinases (Chen et al., 1999, PMID: 1047253 [82]). Regulates the cytoskeleton and dendrite formation (de Anda et al., 2012, PMID: 22683681 [83]; Nourbakhsh et al., 2021, PMID: 34879262 [84]). Loss induces ASD-relevant phenotypes in mice (Richter et al., 2019, PMID: 29467497 [85]). *MVP:* neuronal function incompletely understood, regulates ERK signalling. Regulates brain morphology and modified anxiety-like behaviour in mice (Kretz et al., 2023, PMID: 37968726 [86]). *KCTD13*: Regulates neuronal development (Kizner et al., 2020, PMID: 31402430 [87]) and excitatory neurotransmission (Gu et al., 2023, PMID: 37142655 [88]).
15q13.3 deletion/duplication	∼6 genes including Cholinergic receptor nicotinic alpha 7 subunit (*CHRNA7*), OTT-domain containing protein 7A (*OTUD7A*), Transient receptor potential cation channel subfamily M member 1 (*TRPM1*)	Deletion: ∼10% affected individuals have an ASD diagnosis (Lowther et al., 2015, PMID: 25077648 [89]; Breakpoint BP4-BP5).	*CHRNA7*: Nicotinic acetylcholine receptor subunit, modulates glutamatergic (Stone, 2021, PMID: 34111447 [90]) and GABAergic (Lin et al., 2014, PMID: 24983521 [91]) transmission. *OTUD7A*: putative deubiquitinating enzyme that localises to dendritic spines. Regulates glutamatergic synapse development (Kozlova et al., 2022, PMID: 35931052 [92]) and deletion induces ASD-relevant behavioural phenotypes in mice (Yin et al., 2018, PMID: 29395075 [93]). *TRPM1*: divalent cation (Ca^2+^, Mg^2+^, Zn^2+^) permeable channel, potentially interacts with glutamate mGluR6 in neurons (Shen et al., 2012, PMID: 22586107 [94]) and deletion induces ASD-relevant phenotypes in mice (Hori et al., 2021, PMID: 33785025 [95]).
22q11.2 deletion/duplication	∼90 genes (∼40 protein coding expressed in brain) including T-Box transcription factor 1 *(TBX1)*, *DiGeorge syndrome critical region 8 (DGCR8)*, *Proline dehydrogenase (PRODH)*	∼2.95 HR (Olsen et al., 2018, PMID: 29886042 [96])	*TBX1*: encodes transcription factors regulating developmental processes (Papaioannou et al., 2014, PMID: 25294936 [97]). Heterozygous deletion impairs myelination, reduces postnatal progenitor cells in hippocampus and impairs cognitive flexibility (Hiramoto et al., 2022, PMID : 34737458 [98]). *DGCR8*: Regulates microRNA biogenesis (Burger and Gullerova, 2015, PMID: 26016561 [99]). Influences brain development and heterozygous deletion impacts on GABAergic signalling and neuronal network plasticity (Amin et al., 2017, PMID: 29146941 [100]). *PRODH*: Regulates proline metabolism. Prodh deficient mice show altered glutamate and GABA function (Paterlini et al., 2005, PMID: 16234811 [101]) and Prodh depletion impacts on neuronal morphology (Yao et al., 2024, PMID: 37815900 [102]).

A selection of genes and CNVs associated with an increased risk of developing ASD, with a focus on those with a large number of cases that have currently been relatively well characterised in rodent models.

a A few select genes of interest shown for polygenic CNVs only. A more extensive list of risk genes and CNVs is available at http://gene.sfari.org.

### Genetic rodent models relevant to ASD

**Figure 1.**
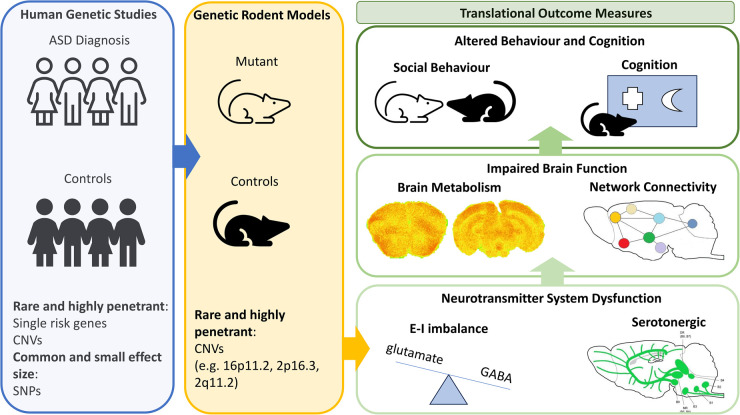
Translational rodent models based on genetic risk factors for ASD provide new mechanistic insight into the aetiological basis of the disorder. Risk genes identified in human genetic studies have identified common variants of small effect (such as SNPs) and rare highly penetrant risk genes and CNVs. Rodent models based on risk CNVs identified in human studies have shown these risk genes impair neurotransmitter system function, particularly in terms of E-I (glutamate-GABA) balance, and potentially in serotonin system function. This neurotransmitter system dysfunction contributes to abnormal brain metabolism/function and brain network connectivity, which underscores abnormal behaviour and cognition, including deficits in social and executive function.

Due to their small effect size, genetic rodent models based on risk SNPs in common gene variants, such as those identified as being associated with ASD by GWAS, have not yet been prioritised. To ensure translatability, preclinical rodent models with high construct validity are required. In the context of risk SNPs in common gene variants, such genetic models would optimally be based on multiple risk gene polymorphisms, which is technically challenging. Future preclinical work in ASD will no doubt benefit from the development of SNP-based, polygenic risk gene models with high construct validity. Instead, preclinical rodent models for ASD based on single risk genes and CNVs with high penetrance have been prioritised, in part due to the technical capability of generating these models but also due to the predicted pronounced impact of these genetic changes in terms of neurobiology and translational phenotypes (Figure 1). One potential limitation of this approach is that the observations made may not be more broadly translational to the general ASD population, due to the relative rarity of these mutations. However, the hope is that key aetiological mechanisms can be identified and prioritised for drug development by integrating observations across multiple CNV and risk gene models. In addition, these models offer the advantage that they have high construct validity for individuals who harbour the specific genetic mutation, aligning with a personalised-medicine focus for drug development and the opportunity to develop new interventions for individuals harbouring these mutations. In addition, many CNVs affect multiple genes (e.g. 16p11.2 deletion/duplication) and so align with the polygenic risk basis of ASD, which potentially confers a higher overall construct validity and generalisability to these models in comparison with those based on single risk gene mutations.

### Risk mechanism insights from CNV models

Utilising rodent models based on risk CNVs has been fundamental in improving our understanding of the key biological mechanisms implicated in the neurobiology of ASD. Often, surprising and unexpected alterations in neurobiology emerge from these CNVs, particularly at the systems-level, due to their complex effects on neurodevelopmental processes and trajectories. This highlights the essential utility of risk CNV rodent models, and other risk gene models, in addressing the challenges of understanding ASD aetiology and in the drug development process for these disorders. Here we review a range of recent insights gained from three rodent models based on CNV deletions associated with an increased risk of developing ASD, with a focus on the impact at the systems-level, including the impact on cognition, behaviour, brain network connectivity and neurotransmitter system function.

### 16p11.2 deletion

One of the first and most common CNVs associated with ASD is located at 16p11.2, with both deletions and duplications conferring increased ASD risk [19–21]. Interestingly, there is evidence for clinical heterogeneity between 16p11.2 deletion and duplication carriers, with duplication carriers more likely to be diagnosed with attention deficit hyperactivity disorder (ADHD) and psychotic symptoms than individuals carrying a 16p11.2 deletion [22]. This suggests that there is uncharacterised, mechanistic heterogeneity between deletion and duplication at this locus, that results in differential risk for ADHD and psychotic psychopathology, and potentially to ASD symptomatology, which is yet to be studied in detail. In addition to ASD, 16p11.2 CNVs are associated with intellectual disability and epilepsy [23,24], which may result from disturbed excitation-inhibition (E-I) neurotransmitter balance in the brain, as discussed below.

Mouse models with a 16p11.2 deletion analogous to human carriers, which results in haploinsufficiency of ∼29 genes (including *Taok2*, *Mapk3* and *Mvp*, Table 1) show ASD-relevant behavioural phenotypes including hyperactivity and repetitive circling behaviours [25]. Interestingly, enhanced cognitive abilities have also been shown in 16p11.2 deletion mice in terms of visual attentional processing [26], suggesting that the model reflects both key behavioural deficits and some of the enhanced cognitive abilities reported in individuals with ASD, such as increased visual search ability [27]. In terms of brain network connectivity, there is evidence for the widespread functional brain network dysconnectivity in 16p11.2 deletion mice, that includes abnormal prefrontal cortex (PFC) connectivity, which parallels that seen in human individuals with 16p11.2 deletion and those with ASD [26,28]. In addition, there is evidence for widespread E-I neurotransmitter system imbalance in 16p11.2 deletion mice [26,29,30] with some behavioural (activity and social) deficits corrected by the E-I neurotransmission modulator N-acetyl cysteine [31]. Moreover, there is also emerging evidence for serotonergic dysfunction as a consequence of 16p11.2 deletion, with the hyperactivity and social deficits in these animals corrected by the 5-HT_1B/1D/1F_ receptor agonist eletriptane [31], sociability deficits reversed by MDMA and the 5-HT_1B_ agonist CP-94,253 [32], and performance in the forced swim test rescued by the 5-HT_2A_ receptor antagonist volinanserin (MDL 100 907) [33]. Despite these pharmacological observations, the cellular and molecular basis of serotonin (5-hydroxytryptamine, 5-HT) system dysfunction in this model has not yet been characterised. This would certainly be of interest, to further understand its role in ASD symptom risk.

### 2p16.3 deletion

2p16.3 deletions, involving heterozygous deletion of the *NRXN1* gene, substantially increase the risk of developing ASD [20,34,35]. The vast majority of 2p16.3 deletions identified impact on the longer NRXN1α isoform, while leaving the shorter NRXN1β isoform intact, resulting in heterozygous NRXN1α deletion. NRXN1α is a presynaptic protein that interacts with a diverse range of post-synaptic binding partners to regulate synaptic maturation and efficacy [36,37].

Mouse models based on decreased NRXN1α expression have identified a range of behavioural phenotypes relevant to ASD, and to human individuals with the 2p16.3 deletion. This includes delayed development, abnormal communication (ultrasonic vocalisations) and impaired memory and executive cognition [38–40]. In addition, *Nrxn1*α heterozygous (Hz) deletion in mice induces deficits in brain metabolism and network connectivity that have translational relevance to the alterations seen in individuals with ASD, including reduced PFC metabolism and abnormal PFC connectivity [40,41]. Evidence from *Nrxn1α* Hz mice also supports disturbed balance in terms of excitatory (glutamatergic) and inhibitory (GABAergic) neurotransmitter system function in the brain [40], and the regulation of E-I balance by Nrxn1α is supported in other mouse models [42,43]. Altered E-I balance in *Nrxn1*α Hz mice parallels the disturbed E-I balance reported in individuals with ASD, and data from cultured human cortical neurons from ASD patients with *NRNX1* heterozygous deletion supports neuron hyperexcitability and abnormal glutamatergic function [44]. Emerging data from *Nrxn1*α Hz mice also support potentially dysfunctional serotonergic signalling [40], which may parallel dysfunctional serotonin activity in individuals with ASD [45]. More work is needed to define the nature of serotonin system dysfunction that results from *Nrxn1*α heterozygosity and whether serotonergic drugs can correct some of the behavioural phenotypes observed. This could include testing some of the serotonergic drugs that have previously been found to be beneficial in 16p11.2 deletion mice.

### 22q11.2 deletions

Genetic deletions at 22q11.2, resulting in DiGeorge (velocardiofacial) syndrome, have been relatively well characterised and are associated with increased risk of developing ASD, and a range of other brain disorders, including schizophrenia [46,47]. The typical ∼3.0 Mb 22q11.2 deletion region contains ∼90 genes, including 46 protein coding genes with confirmed expression in the human brain [48].

Mouse models that either partially or fully replicate the ∼3.0 Mb deletion seen in most patients, have been generated [49]. These mouse models demonstrate a range of behavioural phenotypes and cognitive deficits which parallel those seen in individuals with ASD, including impaired social memory and circadian rhythms [49–51]. In terms of brain network connectivity, abnormal PFC connectivity has been reported in 2q11.2 mouse models [52,53] and is also found in humans with the 22q11.2 deletion [54]. Studies in both humans with the deletion and 2p11.2 deletion mouse models also support disturbed E-I neurotransmitter system balance because of the deletion, with alterations in both glutamate and GABAergic neurotransmission supported [55–57], although effects on glutamate are not always found in patients [58]. While several studies have characterised the impact of 22q11.2 deletion on dopaminergic function in the brain, given that the dopamine degrading enzyme Catechol-*O*-methyltransferase is coded for in the 22q11.2 region and its independent association with schizophrenia (reviewed in [59]), very few studies have characterised the impact on serotoninergic function. However, lower urine serotonin levels have been reported in individuals with 2q11.2 deletion, which were found to positively correlate with IQ [60] and recent evidence supports a potential positive effect of long-term selective serotonin reuptake inhibitor (SSRI) treatment on cognition in children and adolescents with the deletion [61]. Thus, further study on the impact of the 2q11.2 deletion on serotonin neurotransmitter system function is certainly warranted.

## Summary: integrating observations across multiple risk gene models to identify risk mechanism and drug target prioritisation

Observations across the CNV models outlined above support a role for altered synaptic function, E-I balance and potential serotoninergic dysfunction as key alterations underlying ASD symptomatology. While E-I imbalance has been relatively well characterised, further work on the potential role of disturbed serotonin system function in these CNV models is needed. While E-I balance is more studied, important gaps in our understanding of how E-I balance is altered across these models remain, particular in terms of (i) the brain regions most affected, (ii) the molecular aspects of glutamate and GABA neurotransmitter system function most impacted, (iii) the relationship of these changes to functional outcomes, and (iv) the conserved nature of these specific changes across the different models. More systematic, granular, and integrated work across these models is required to further elucidate the conserved changes in E-I function and their relationship to behavioural and cognitive outcomes. This seems particularly important given evidence supporting the potential efficacy of drugs targeting E-I balance for some ASD symptoms [62,63].

The emerging data supporting serotonergic dysfunction across these models is also of particular interest given the diverse data supporting serotonergic dysfunction in people with ASD and in other ASD-relevant animal models. Observations in humans with an ASD diagnosis include reduced serotonin-reuptake transporter (SERT) availability, lower expression of the 5-HT degrading enzyme mono-amine oxidase A (MAOA) and complex, subtype-dependent alterations in 5-HT receptor expression (reviewed in [45]). In addition, data supporting the ability of drugs targeting the serotonergic system to relieve some ASD symptoms, particularly SSRIs that block SERT [64,65], also implicate dysfunction of this neurotransmitter system in ASD, although positive effects are not always found [66] and the evidence for some ASD symptom domains is very limited. The serotonin system, a monoamine, modulatory neurotransmitter system, originates from cells located in the raphé nuclei of the brainstem with extensive projections to the forebrain and brainstem in both humans and rodents (Figure 1) [67,68]. Thus, the serotonin system innervates and modulates activity in a range of brain regions known to be dysfunctional in ASD, including the PFC and hippocampus [69,70]. The serotonin system utilises a diverse range of receptors, ordered into seven families (5-HT_1–7_), in its signalling, with some acting primarily as post-synaptic effectors (e.g. 5-HT_2_) and others with functions as both post-synaptic effectors and autoreceptors (e.g. 5-HT_1A_) that modulate serotonin release [71]. A range of receptor agonists and antagonists, with variable selectivity, are available to target these receptors, supporting the tractability of the serotonin system as a therapeutic target in ASD (https://www.guidetopharmacology.org [72]). The serotonin system also regulates a wide range of behavioural and cognitive processes relevant to ASD symptomatology, including vulnerability to social stress [73], repetitive/compulsive behaviours [74] and the regulation of executive function [75], further supporting the potential of targeting the serotonin system to relieve these symptoms in ASD. Given the emerging data supporting serotonin system dysfunction in the CNV models considered in this review, further work is required to understand the changes present and the specific molecular aspects of serotonin system signalling that are of putative therapeutic value in these models and ASD.

Converging evidence across multiple CNV models also supports the PFC as an important locus of brain network dysfunction, paralleling PFC dysfunctional connectivity in ASD [76]. This PFC dysfunction likely contributes to the higher-order cognitive, emotional, motor and interoceptive processing alterations experienced by individuals with ASD. Thus, pharmacological interventions that aim to restore PFC function and connectivity to alleviate these symptoms are of particular interest. The above consideration of the available literature highlights the utility of combining observations made across multiple genetic models relevant to ASD in identifying key aetiological mechanisms and prioritising these for drug validation. This integration is particularly important given that penetrance for any given CNV is incomplete and the prevalence of ASD symptom heterogeneity in both individuals harbouring these CNVs and those with an ASD diagnosis. While conserved changes across models may be insightful in terms of prioritising targets for the drug development process, divergences between models are also of interest. There is considerable heterogeneity in symptomatology across individuals with an ASD diagnosis, that likely results in part from underlying genetic risk and the resultant changes in neurobiology. This is very poorly understood. Thus, identifying mechanisms that diverge across mouse models may be useful in informing the neurobiological basis of symptom heterogeneity in ASD.

## Future directions

Insight into the risk genes that underly ASD has provided valuable new information into the aetiological mechanisms that underlie the condition and have identified novel targets for drug development aimed at improving symptoms. However, several challenges remain, including the generalisability of observations made in models based on rare single risk genes and CNVs. While integrating information from multiple models to identify conserved mechanisms may overcome these challenges, particularly in the context of prioritising the focus of future drug development for ASD, developing new genetic rodent models with high construct validity and broad generalisability, based on multiple risk gene variants of small effect, will also be important. Integrating existing and new genetic models with established environmental risk factor manipulations will also be useful, providing new insight into mechanisms that regulate genetic penetrance. In addition, future work systematically characterising the diversity of biological mechanisms underlying symptom heterogeneity in ASD, and the relevance of different risk factor models to these, is required.

## Perspectives

- Understanding the biological mechanisms underlying the risk of developing ASD is an essential step in developing novel therapeutics. Human genetic studies and rodent models based on rare but highly penetrant genetic risk factors have been fundamental in improving our understanding of the mechanistic basis of ASD.
- Integrating information from multiple rodent transgenic risk models, including CNV models, has identified key aetiological mechanisms in ASD risk. Observations made in CNV models support altered synaptic function, E-I neurotransmitter balance and abnormal brain network connectivity as key ASD risk mechanisms.
- Emerging evidence from CNV models also supports serotonergic dysfunction in ASD, but this remains underexplored. To date, polygenic common gene variant, environmental and modifiable risk factor preclinical models have been relatively under-utilised. Future studies using these models will likely provide valuable new insight into the mechanistic basis of ASD and will be useful in the drug development and validation process.

## Open Access

Open access for this article was enabled by the participation of Lancaster University in an all-inclusive *Read & Publish* agreement with Portland Press and the Biochemical Society under a transformative agreement with JISC.

## Abbreviations

ADHD: attention deficit hyperactivity disorder
ASD: autism spectrum disorder
CNV: copy number variant
E-I: excitation-inhibition
GWAS: Genome Wide Association Studies
PFC: prefrontal cortex
MAOM: monoamine oxidase a
SERT: serotonin-reuptake transporter
SSRI: selective serotonin reuptake inhibitor

## References

[1] American Psychiatric Association. (2013) Diagnostic and Statistical Manual of Mental Disorders: DSM-5, 5th edn., American Psychiatric Publishing, Washington, DC

[2] Maenner, M.J., Warren, Z., Williams, A.R., Amoakohene, E., Bakian, A.V., Bilder, D.A. et al. (2023) Prevalence and characteristics of autism spectrum disorder among children aged 8 years — autism and developmental disabilities monitoring network, 11 sites, United States, 2020. MMWR Surveill. Summ. 72, 1–14 10.15585/mmwr.ss7202a1 PMC10042614 36952288

[3] Sandin, S., Lichtenstein, P., Kuja-Halkola, R., Hultman, C., Larsson, H. and Reichenberg, A. (2017) The heritability of autism spectrum disorder. JAMA 318, 1182–1184 10.1001/jama.2017.12141 28973605 PMC5818813

[4] Xie, S., Karlsson, H., Dalman, C., Widman, L., Rai, D., Gardner, R.M. et al. (2020) The familiar risk of autism spectrum disorder with and without intellectual disability. Autism Res. 13, 2242–2250 10.1002/aur.2417 33103358 PMC7821228

[5] Wu, S., Wu, F., Ding, Y., Hou, J., Bi, J. and Zhang, Z. (2017) Advanced parental age and autism risk in children: a systematic review and meta-analysis. Acta Psychiatr. Scand. 135, 29–41 10.1111/acps.12666 27858958

[6] Rosen, B.N., Lee, B.K., Lee, N.L., Yang, Y. and Burstyn, I. (2015) Maternal smoking and autism spectrum disorder: a meta-analysis. J. Autism Dev. Disord. 45, 1689–1698 10.1007/s10803-014-2327-z 25432101

[7] Tioleco, N., Silberman, A.E., Stratigos, K., Banerjee-Basu, S., Spann, M.N., Whitaker, A.H. et al. (2021) Prenatal maternal infection and risk of autism in offspring: a meta-analysis. Autism Res. 14, 1296–1316 10.1002/aur.2499 33720503

[8] Hudac, C.M., Bove, J., Barber, S., Duyzend, M., Wallace, A., Lese Martin, C. et al. (2020) Evaluating heterogeneity in ASD symptomatology, cognitive ability and adaptive functioning among 16p11.2 CNV carriers. Autism Res. 13, 1300–1310 10.1002/aur.2332 32597026

[9] Andersen, C.H., Thomsen, P.H., Nohr, E.A. and Lemcke, S. (2018) Maternal body mass index before pregnancy as a risk factor for ADHD and autism in children. Eur. Child Adolesc. Psychiatry 27, 139–148 10.1007/s00787-017-1027-6 28712019

[10] Gialloreti, L.E., Mazzone, L., Benvenuto, A., Fasano, A., Alcon, A.G., Kraneveld, A. et al. (2019) Risk and protective environmental factors associated with autism spectrum disorder: evidenced-based principles and recommendations. J. Clin. Med. 8, 217 10.3390/jcm8020217 30744008 PMC6406684

[11] Kheirouri, S. and Alizadeh, M. (2020) Maternal excessive gestational weight gain as a risk factor for autism spectrum disorder in offspring: a systematic review. BMC Pregnancy Childbirth 20, 645 10.1186/s12884-020-03324-w 33092575 PMC7579946

[12] Matias, S.L., Pearl, M., Lyall, K., Croen, L.A., Kral, T.V.E., Fallin, D. et al. (2021) Maternal prepregnancy weight and gestational weight gain in association with autism and developmental disorders in offspring. Obesity 29, 1554–1564 10.1002/oby.23228 34347372 PMC9186321

[13] Grove, J., Ripke, S., Als, T.D., Mattheisen, M., Walters, R.K., Won, H. et al. (2019) Identification of common genetic risk variants for autism spectrum disorder. Nat. Genet. 51, 431–444 10.1038/s41588-019-0344-8 30804558 PMC6454898

[14] Matoba, N., Liang, D., Sun, H., Aygun, N., McAfee, J.C. and Davis, J.E. (2020) Common genetic risk variants identified in the SPARK cohort support DDHD2 as a candidate risk gene for autism. Transl. Psychiatry 10, 265 10.1038/s41398-020-00953-9 32747698 PMC7400671

[15] Narita, A., Nagai, M., Mizuno, S., Ogishima, S., Tamiya, G. and Ueki, M. (2020) Clustering by phenotype and genome-wide association study in autism. Transl. Psychiatry 10, 290 10.1038/s41398-020-00951-x 32807774 PMC7431539

[16] Sebat, J., Lakshmi, B., Malhotra, D., Troge, J., Lese-Martin, C., Walsh, T. et al. (2007) Strong association of de novo copy number mutations with autism. Science 316, 445–444 10.1126/science.1138659 17363630 PMC2993504

[17] Leppa, V.M., Kravitz, S.N., Martin, C.L., Andrieux, J., Le Caignec, C., Martin-Coignard, D. et al. (2016) Rare inherited and de novo CNVs reveal complex contributions to ASD risk in multiplex families. Am. J. Hum. Genet. 99, 540–554 10.1016/j.ajhg.2016.06.036 27569545 PMC5011063

[18] Torrico, B., Fernàndez-Castillo, N., Hervás, A., Mila, M., Salgado, M., Rueda, I. et al. (2015) Contribution of common and rare variants of the PTCHD1 gene to autism spectrum disorders and intellectual disability. Eur. J. Hum. Genet. 23, 1694–1701 10.1038/ejhg.2015.37 25782667 PMC4795195

[19] Weiner, D.J., Ling, S., Erdin, S., Tai, D.J.C., Yadav, R., Grove, J. et al. (2022) Statistical and functional convergence of common and rare genetic influences on autism at chromosome 16p. Nat. Genet. 54, 1630–1639 10.1038/s41588-022-01203-y 36280734 PMC9649437

[20] Marshall, C.R., Noor, A., Vincent, J.B., Lionel, A.C., Feuk, L., Skaug, J. et al. (2008) Structural variation of chromosomes in autism spectrum disorder. Am. J. Hum. Genet. 82, 477–488 10.1016/j.ajhg.2007.12.009 18252227 PMC2426913

[21] Levy, D., Ronemus, M., Yamrom, B., Lee, Y.-H., Leotta, A., Kendall, J. et al. (2011) Rare de novo and transmitted copy-number variation in autistic spectrum disorders. Neuron 70, 886–897 10.1016/j.neuron.2011.05.015 21658582

[22] Niarchou, M., Chawner, S.J.R.A., Doherty, J.L., Maillard, A.M., Jacquemont, S., Chung, W.K. et al. (2019) Psychiatric disorders in children with 16p11.2 deletion and duplication. Transl. Psychiatry 9, 8 10.1038/s41398-018-0339-8 30664628 PMC6341088

[23] D'Angelo, D., Lebon, S., Chen, Q., Martin-Brevet, S., Green Snyder, L., Hippolyte, L. et al. (2016) Defining the effect of the 16p11.2 duplication on cognition, behavior, and medical comorbidities. JAMA Psychiatry 73, 20–30 10.1001/jamapsychiatry.2015.2123 26629640 PMC5894477

[24] Moufawad El Achkar, C., Rosen, A., Kessler, S.K., Steinman, K.J., Spence, S.J., Ramocki, M. et al. (2022) Clinical characteristics of seizures and epilepsy in individuals with recurrent deletions and duplications in the 16p11.2 region. Neurol. Genet. 8, e200018 10.1212/NXG.0000000000200018 36531974 PMC9756306

[25] Portmann, T., Yang, M., Mao, R., Panagiotakos, G., Ellegood, J., Dolen, G. et al. (2014) Behavioral abnormalities and circuit defects in the basal ganglia of a mouse model of 16p11.2 deletion syndrome. Cell Rep. 7, 1077–1092 10.1016/j.celrep.2014.03.036 24794428 PMC4251471

[26] Openshaw, R.L., Thomson, D.M., Bristow, G.C., Mitchell, E.J., Pratt, J.A., Morris, B.J. et al. (2023) 16p11.2 deletion mice exhibit compromised fronto-temporal connectivity, GABAergic dysfunction and enhanced attentional ability. Commun. Biol. 6, 577 10.1038/s42003-023-04891-2 37225770 PMC10209099

[27] Kaldy, Z., Giserman, I., Carter, A.S. and Blaser, E. (2016) The mechanisms underlying the ASD advantage in visual search. J. Autism Dev. Disord. 46, 1513–1527 10.1007/s10803-013-1957-x 24091470 PMC3976471

[28] Bertero, A., Liska, A., Pagani, M., Parolisi, R., Esteban Masferrer, M. and Gritti, M. (2018) Autism-associated 16p11.2 microdeletion impairs prefrontal functional connectivity in mouse and human. Brain 141, 2055–2065 10.1093/brain/awy111 29722793

[29] Lu, H.-C., Mills, A.A. and Tian, D. (2018) Altered synaptic transmission and maturation of hippocampal CA1 neurons in a mouse model of human chr16p11.2 microdeletion. J. Neurophysiol. 119, 1005–1018 10.1152/jn.00306.2017 29212915 PMC7864230

[30] Tai, D.J.C., Razaz, P., Erdin, S., Gao, D., Wang, J., Nuttle, X. et al. (2022) Tissue- and cell-type-specific molecular and functional signatures of 16p11.2 reciprocal genomic disorder across mouse brain and human neuronal models. Am. J. Hum. Genet. 109, 1789–1813 10.1016/j.ajhg.2022.08.012 36152629 PMC9606388

[31] Mitchell, E.J., Thomson, D.M., Openshaw, R.L., Bristow, G.C., Dawson, N., Pratt, J.A. et al. (2020) Drug-responsive autism phenotypes in the 16p11.2 deletion mouse model: a central role for gene-environment interactions. Sci. Rep. 10, 12303 10.1038/s41598-020-69130-8 32704009 PMC7378168

[32] Walsh, J.J., Llorach, P., Cardozo Pinto, D.F., Wenderski, W., Christoffel, D.J., Salgado, J.S. et al. (2021) Systematic enhancement of serotonin signaling reverses social deficits in multiple mouse models for ASD. Neuropsychopharmacology 46, 2000–2010 10.1038/s41386-021-01091-6 34239048 PMC8429585

[33] Panzini, C.M., Ehlinger, D.G., Alchahin, A.M., Guo, Y. and Commons, K.G. (2017) 16p11.2 deletion syndrome mice perseverate with active coping response to acute stress - rescue by blocking 5-HT2A receptors. J. Neurochem. 2017, 708–721 10.1111/jnc.14227 PMC5729115 28948999

[34] Matsunami, N., Hadley, D., Hensel, C.H., Christensen, G.B., Kim, C., Frackelton, E. et al. (2013) Identification of rare recurrent copy number variants in high-risk autism families and their prevalence in a large ASD population. PLoS One 8, e52239 10.1371/journal.pone.0052239 23341896 PMC3544904

[35] Wang, J., Gong, J., Li, L., Chen, Y., Liu, L., Gu, H. et al. (2018) Neurexin gene family variants as risk factors for autism spectrum disorder. Autism Res. 11, 37–43 10.1002/aur.1881 29045040

[36] Gomez, A.M., Traunmuller, L. and Scheiffele, P. (2021) Neurexins: molecular codes for shaping neuronal synapses. Nat. Rev. Neurosci. 22, 137–151 10.1038/s41583-020-00415-7 33420412 PMC7612283

[37] Sudhof, T.C. (2017) Synaptic Neurexin complexes: a molecular code for the logic of neural circuits. Cell 171, 745–769 10.1016/j.cell.2017.10.024 29100073 PMC5694349

[38] Dachtler, J., Ivorra, J.L., Rowland, T.E., Lever, C., Rodgers, R.J. and Clapcote, S.J. (2015) Heterozygous deletion of α-neurexin I or α-neurexin II results in behaviors relevant to autism and schizophrenia. Behav. Neurosci. 129, 765–776 10.1037/bne0000108 26595880 PMC4655861

[39] Armstrong, E.C., Caruso, A., Servadio, M., Andreae, L.C., Trezza, V., Scattoni, M.L. et al. (2020) Assessing the developmental trajectory of mouse models of neurodevelopmental disorders: social and communication deficits in mice with *Neurexin 1α* deletion. Genes, Brain Behav. 19, e12630 10.1111/gbb.12630 31823470

[40] Hughes, R.B., Whittingham-Dowd, J., Clapcote, S.J., Broughton, S.J. and Dawson, N. (2022) Altered medial prefrontal cortex and dorsal raphé activity predict genotype and correlate with abnormal learning behavior in a mouse model of autism-associated 2p16.3 deletion. Autism Res. 15, 614–627 10.1002/aur.2685 35142069 PMC9303357

[41] Hughes, R.B., Whittingham-Dowd, J., Simmons, R.E., Clapcote, S.J., Broughton, S.J. and Dawson, N. (2020) Ketamine restores thalamic-prefrontal cortex functional connectivity in a mouse model of neurodevelopmental disorder-associated 2p16.3 deletion. Cereb. Cortex 30, 2358–2371 10.1093/cercor/bhz244 31812984 PMC7175007

[42] Uchigashima, M., Konno, K., Demchak, E., Cheung, A., Watanabe, T. and Keener, D.G. (2020) Specific Neuroligin-3-αNeurexin-1 signaling regulates GABAergic synaptic function in mouse hippocampus. eLife 9, e59545 10.7554/eLife.59545 33355091 PMC7758064

[43] Lu, H., Zuo, L., Roddick, K.M., Zhang, P., Oku, S., Garden, J. et al. (2023) Alternative splicing and heparan sulfation converge on neurexin-1 to control glutamatergic transmission and autism-related behaviors. Cell Rep. 42, 112714 10.1016/j.celrep.2023.112714 37384525

[44] Avazzadeh, S., Quinlan, L.R., Reilly, J., McDonagh, K., Jalali, A., Wang, Y. et al. (2021) *NRXN1*α+/- is associated with increased excitability in ASD iPSC-derived neurons. BMC Neurosci. 22, 56 10.1186/s12868-021-00661-0 34525970 PMC8442436

[45] Rodnyy, A.Y., Kondaurova, E.M., Tsybko, A.S., Popova, N.K., Kudlay, D.A. and Naumenko, V.S. (2024) The brain serotonin system in autism. Rev. Neurosci. 35, 1–20 10.1515/revneuro-2023-0055 37415576

[46] Fiksinski, A.M., Breetvelt, E.J., Duijff, S.N., Bassett, A.S., Kahn, R.S. and Vorstman, J.A.S. (2017) Autism spectrum and psychosis risk in the 22q11.2 deletion syndrome: findings from a prospective longitudinal study. Schizophr. Res. 188, 59–62 10.1016/j.schres.2017.01.032 28119035 PMC5522359

[47] Schneider, M., Debbane, M., Bassett, A.S., Chow, E.W.C., Fung, W.L.A., van den Bree, M.B.M. et al. (2014) Psychiatric disorders from childhood to adulthood in 22q11.2 deletion syndrome: results from the international consortium on brain and behavior in 22q11.2 deletion syndrome. Am. J. Psychiatry 171, 627–639 10.1176/appi.ajp.2013.13070864 24577245 PMC4285461

[48] Guna, A., Butcher, N.J. and Bassett, A.S. (2015) Comparative mapping of the 22q11.2 deletion region and the potential of simple model organisms. J. Neurodev. Disord. 7, 18 10.1186/s11689-015-9113-x 26137170 PMC4487986

[49] Saito, R., Koebis, M., Nagai, T., Shimizu, K., Liao, J., Wulaer, B. et al. (2020) Comprehensive analysis of a novel mouse model of the 22q11.2 deletion syndrome: a model with the most common 3.0-Mb deletion at the human 22q11.2 locus. Transl. Psychiatry 10, 35 10.1038/s41398-020-0723-z 32066675 PMC7026107

[50] Piskorowski, R.A., Nasrallah, K., Diamantopoulou, A., Mukai, J., Hassan, S.I., Siegelbaum, S.A. et al. (2016) Age-dependent specific changes in area CA2 of the hippocampus and social memory deficit in a mouse model of the 22q11.2 deletion syndrome. Neuron 89, 163–176 10.1016/j.neuron.2015.11.036 26748091 PMC4706988

[51] Tripathi, A., Spedding, M., Schenker, E., Didriksen, M., Cressant, A. and Jay, T.M. (2020) Cognition- and circuit-based dysfunction in a mouse model of 22q11.2 microdeletion syndrome: effects of stress. Transl. Psychiatry 10, 41 10.1038/s41398-020-0687-z 32066701 PMC7026063

[52] Gass, N., Peterson, Z., Reinwald, J., Sartorius, A., Weber-Fahr, W., Sack, M. et al. (2021) Differential resting-state patterns across networks are spatially associated with *Comt* and *Trmt2a* gene expression patterns in a mouse model of 22q11.2 deletion. Neuroimage 243, 118520 10.1016/j.neuroimage.2021.118520 34455061 PMC9063447

[53] Sigurdsson, T., Stark, K.L., Karayiorgou, M., Gogos, J.A. and Gordon, J.A. (2010) Impaired hippocampal-prefrontal synchrony in a genetic mouse model of schizophrenia. Nature 464, 763–767 10.1038/nature08855 20360742 PMC2864584

[54] Zoller, D., Sandini, C., Karahanoglu, F.I., Padula, M.C., Schaer, M., Eliez, S. et al. (2019) Large-scale brain network dynamics provide a measure of psychosis and anxiety in 22q11.2 deletion syndrome. Biol. Psychiatry Cogn. Neurosci. Neuroimaging 4, 881–892 10.1016/j.bpsc.2019.04.004 31171499

[55] Al-Absi, A.R., Qvist, P., Okujeni, S., Khan, A.R., Glerup, S., Sanchez, C. et al. (2020) Layers II/III of prefrontal cortex in Df(h22q11)/+ mouse model of the 22q11.2 deletion display loss of parvalbumin interneurons and modulation of neuronal morphology and excitability. Mol. Neurobiol. 57, 4978–4988 10.1007/s12035-020-02067-1 32820460

[56] Al-Absi, A.R., Thambiappa, S.K., Khan, A.R., Glerup, S., Sanchez, C., Landau, A.M. et al. (2022) Df(h22q11)/+ mouse model exhibits reduced binding levels of GABA_A_ receptors and structural and functional dysregulation in the inhibitory and excitatory networks of hippocampus. Mol. Cell Neurosci. 122, 103769 10.1016/j.mcn.2022.103769 35988854

[57] Mancini, V., Saleh, M.G., Delavari, F., Bagautdinova, J. and Eliez, S. (2023) Excitatory/Inhibitory imbalance underlies hippocampal atrophy in individuals with 22q11.2 deletion syndrome with psychotic symptoms. Biol. Psychiatry 94, 569–579 10.1016/j.biopsych.2023.03.021 37011759

[58] Rogdaki, M., Hathway, P., Gudbrandsen, M., McCutcheon, R.A., Jauhar, S., Daly, E. et al. (2019) Glutamatergic function in a genetic high-risk group for psychosis: a proton magnetic resonance spectroscopy study in individuals with 22q11.2 deletion. Eur. Neuropsychopharmacol. 29, 1333–1342 10.1016/j.euroneuro.2019.09.005 31648854

[59] Zinkstok, J.R., Boot, E., Bassett, A.S., Hiroi, N., Butcher, N.J., Vingerhoets, C. et al. (2019) Neurobiological perspective of 22q11.2 deletion syndrome. Lancet Psychiatry 6, 951–960 10.1016/S2215-0366(19)30076-8 31395526 PMC7008533

[60] Evers, L.J., Curfs, L.M., Bakker, J.A., Boot, E., da Silva Alves, F., Abeling, N. et al. (2014) Serotonergic, noradrenergic and dopaminergic markers are related to cognitive function in adults with 22q11 deletion syndrome. Int. J. Neuropsychopharmacol. 17, 1159–1165 10.1017/S1461145714000376 24713114

[61] Mancini, V., Maeder, J., Bortolin, K., Schneider, M., Schaer, M. and Eliez, S. (2021) Long-term effects of early treatment with SSRIs on cognition and brain development in individuals with 22q11.2 deletion syndrome. Transl. Psychiatry 11, 336 10.1038/s41398-021-01456-x 34052829 PMC8164636

[62] Aman, M.G., Findling, R.L., Hardan, A.Y., Hendren, R.L., Melmed, R.D., Kehinde-Nelson, O. et al. (2017) Safety and efficacy of memantine in children with autism: randomized, placebo-controlled study and open-label extension. J. Child Adolesc. Psychopharmacol. 27, 403–412 10.1089/cap.2015.0146 26978327 PMC5510039

[63] Karahmadi, M., Tarrahi, M.J., Vatankhah Ardestani, S.S., Omranifard, V. and Farzaneh, B. (2018) Efficacy of memantine as adjunct therapy for autism spectrum disorder in children aged <14 years. Adv. Biomed. Res. 7, 131 10.4103/abr.abr_100_18 30320040 PMC6176686

[64] Liang, S.-C., Sun, C.-K., Fan, H.-Y., Chung, W., Ruu-Fen, T., Hung, K.-C. et al. (2022) Therapeutic effect of antidepressants for global improvement and subdomain symptoms of autism spectrum disorder: a systematic review and meta-analysis. J. Psychiatry Neurosci. 47, E299–E310 10.1503/jpn.210191 35948343 PMC9377548

[65] Williams, K., Brignell, A., Randall, M., Silove, N. and Hazell, P. (2013) Selective serotonin reuptake inhibitors (SSRIs) for autism spectrum disorders (ASD). Cochrane Database Syst. Rev. 8, CD004677 10.1002/14651858.CD004677.pub3 PMC11990048 23959778

[66] Zhou, M.S., Nasir, M., Farhat, L.C., Kook, M., Artukoglu, B.B. and Bloch, M.H. (2021) Meta-analysis: pharmacologic treatment of restrictive and repetitive behaviours in autism spectrum disorder. J. Am. Acad. Child Adolesc. Psychiatry 60, 35–45 10.1016/j.jaac.2020.03.007 32387445

[67] Awashthi, J.R., Tamada, K., Overton, E.T.N. and Takumi, T. (2021) Comprehensive topographical map of the serotonergic fibres in the male mouse brain. J. Comp. Neurol. 529, 1391–1429 10.1002/cns.25027 32892368

[68] Beliveau, V., Ganz, M., Feng, L., Ozenne, B., Hojgaard, L., Fisher, P.M. et al. (2017) A high resolution *in vivo* atlas of the human brain's serotonin system. J. Neurosci. 37, 120–128 10.1523/JNEUROSCI.2830-16.2016 28053035 PMC5214625

[69] Bombardi, C., Grandis, A., Pivac, N., Sagud, M., Lucas, G., Chagraoui, A. et al. (2021) Chapter 3 – serotonin modulations of hippocampal functions: from anatomy to neurotherapeutics. Prog. Brain Res. 261, 83–158 10.1016/bs.pbr.2021.01.031 33785139

[70] Puig, M.V. and Gulledge, A.T. (2011) Serotonin and prefrontal cortex function: neurons, networks, and circuits. Mol. Neurobiol. 44, 449–464 10.1007/s12035-011-8214-0 22076606 PMC3282112

[71] Pytliak, M., Vargova, V., Mechirova, A. and Felsoci, M. (2011) Serotonin receptors – from molecular biology to clinical applications. Physiol. Rev. 60, 15–25 10.33549/physiolres.931903 20945968

[72] Harding, S.D., Armstrong, J.F., Faccenda, E., Southan, C., Alexander, S.P.H., Davenport, A.P. et al. (2024) The IUPHAR/BPS guide to pharmacology in 2024. Nucleic Acids Res. 52, D1438–D1449 10.1093/nar/gkad944 37897341 PMC10767925

[73] Zou, W.-J., Song, Y.-L., Wu, M.-Y., Chen, X.-T., You, Q.-L., Yang, Q. et al. (2020) A discrete serotonergic circuit regulates vulnerability to social stress. Nat. Commun. 11, 4218 10.1038/s41467-020-18010-w 32839452 PMC7445164

[74] Alvarez, B.D., Cavazos, C., Morales, C.A., Lopez, S.M. and Amodeo, D.A. (2022) Impact of serotonin receptor modulation on restricted repetitive behaviours. Front. Behav. Neurosci. 16, 1078983 10.3389/fnbeh.2022.1078983 36620862 PMC9816668

[75] Luo, Q., Kanen, J.W., Bari, A., Skandli, N., Langley, C., Knudsen, G.M. et al. (2023) Comparable roles for serotonin in rats and humans for computations underlying flexible decision-making. Neuropsychopharmacology 49, 600–608 10.1038/s41386-023-01762-6 37914893 PMC10789782

[76] Leisman, G., Melillo, R. and Melillo, T. (2023) Prefrontal functional connectivities in autism spectrum disorders: a connectopathic disorder affecting movement, interoception and cognition. Brain Behav. Bull. 198, 65–76 10.1016/j.brainresbull.2023.04.004 37087061

[77] Darnell, J.C., Van Driesche, S.J., Zhang, C., Hung, K.Y., Mele, A., Fraser, C.E. et al. (2011) FMRP stalls ribosomal translocation on mRNAs linked to synaptic function and autism. Cell 146, 247–261 10.1016/j.cell.2011.06.013 21784246 PMC3232425

[78] Tillotson, R. and Bird, A. (2020) The molecular basis of MeCP2 function in the brain. J. Mol. Biol. 432, 1602–1623 10.1016/j.jmb.2019.10.004 31629770

[79] Yuen, R.K., Merico, D., Bookman, M., Howe, J., Thiruvahindrapuram, B., Patel, R.V. et al. (2017) Whole genome sequencing resource identifies 18 new candidate genes for autism spectrum disorder. Nat. Neurosci. 20, 602–611 10.1038/nn.4524 28263302 PMC5501701

[80] Walsh, K. and Bracken, M. (2011) Copy number variation in the dosage-sensitive 16p11.2 interval accounts for only a small proportion of autism incidence: a systematic review and meta-analysis. Genet. Med. 13, 377–384 10.1097/GIM.0b013e3182076c0c 21289514

[81] Boutros, T., Chevet, E. and Metrakos, P. (2008) Mitogen-activated protein (MAP) kinase/MAP kinase phosphatase regulation: roles in cell growth, death and cancer. Pharmacol. Rev. 60, 261–310 10.1124/pr.107.00106 18922965

[82] Chen, Z., Hutchison, M. and Cobb, M.H. (1999) Isolation of the protein kinase TAO2 and identification of its mitogen-activated protein kinase/extracellular signal-regulated kinase kinase binding domain. J. Biol. Chem. 274, 28803–28807 10.1074/jbc.274.40.28803 10497253

[83] de Anda, F.C., Rosario, A.L., Durak, O., Tran, T., Gräff, J., Meletis, K. et al. (2012) Autism spectrum disorder susceptibility gene *TAOK2* affects basal dendrite formation in the neocortex. Nat. Neurosci. 15, 1022–1031 10.1038/nn.3141 22683681 PMC4017029

[84] Nourbakhsh, K., Ferreccio, A.A., Bernard, M.J. and Yadav, S. (2021) TAOK2 is an ER-localized kinase that catalyzes the dynamic tethering of ER to microtubules. Dev. Cell 56, 3321–3333.e5 10.1016/j.devcel.2021.11.015 34879262 PMC8699727

[85] Richter, M., Murtaza, N., Scharrenberg, R., White, S.H., Johanns, O., Walker, S. et al. (2019) Altered TAOK2 activity causes autism-related neurodevelopmental and cognitive abnormalities through RhoA signaling. Mol. Psychiatry 24, 1329–1350 10.1038/s41380-018-0025-5 29467497 PMC6756231

[86] Kretz, P.F., Wagner, C., Mikhaleva, A., Montillot, C., Hugel, S., Morella, I. et al. (2023) Dissecting the autism-associated 16p11.2 locus identifies multiple drivers in neuroanatomical phenotypes and unveils a male-specific role for the major vault protein. Genome Biol. 24, 261 10.1186/s13059-023-03092-8 37968726 PMC10647150

[87] Kizner, V., Naujock, M., Fischer, S., Jager, S., Reich, S., Schlotthauer, I. et al. (2020) CRISPR/Cas9-mediated knockout of the neuropsychiatric risk gene *KCTD13* causes developmental deficits in human cortical neurons derived from induced pluripotent stem cells. Mol. Neurobiol. 57, 616–634 10.1007/s12035-019-0172-1 31402430

[88] Gu, J., Ke, P., Guo, H., Liu, J., Liu, Y., Tian, X. et al. (2023) KCTD13-mediated ubiquitination and degradation of GluN1 regulates excitatory synaptic transmission and seizure susceptibility. Cell Death Differ. 30, 1726–1741 10.1038/s41418-023-01174-5 37142655 PMC10307852

[89] Lowther, C., Costain, G., Stavropoulos, D.J., Melvin, R., Silversides, C.K., Andrade, D.M. et al. (2015) Delineating the 15q13.3 microdeletion phenotype: a case series and comprehensive review of the literature. Genet. Med. 17, 149–157 10.1038/gim.2014.83 25077648 PMC4464824

[90] Stone, T.W. (2021) Relationships and interactions between ionotropic glutamate receptors and nicotinic receptors in the CNS. Neuroscience 468, 321–365 10.1016/j.neuroscience.2021.06.007 34111447

[91] Lin, H., Hsu, F.-C., Baumann, B.H., Coulter, D.A., Anderson, S.A. and Lynch, D.R. (2014) Cortical parvalbumin GABAergic deficits with α7 nicotinic acetylcholine receptor deletion: implications for schizophrenia. Mol. Cell Neurosci. 61, 163–175 10.1016/j.mcn.2014.06.007 24983521 PMC4136487

[92] Kozlova, A., Zhang, S., Kotlar, A.V., Jamison, B., Zhang, H., Shi, S. et al. (2022) Loss of function of *OTUD7A* in the schizophrenia- associated 15q13.3 deletion impairs synapse development and function in human neurons. Am. J. Hum. Genet. 109, 1500–1519 10.1016/j.ajhg.2022.07.001 35931052 PMC9388388

[93] Yin, J., Chen, W., Chao, E.S., Soriano, S., Wang, L., Wang, W. et al. (2018) *Otud7a* knockout mice recapitulate many neurological features of 15q13.3 microdeletion syndrome. Am. J. Hum. Genet. 102, 296–308 10.1016/j.ajhg.2018.01.005 29395075 PMC5985472

[94] Shen, Y., Rampino, M.A., Carroll, R.C. and Nawy, S. (2012) G-protein-mediated inhibition of the Trp channel TRPM1 requires the Gβγ dimer. Proc. Natl Acad. Sci. U.S.A. 109, 8752–8757 10.1073/pnas.1117433109 22586107 PMC3365217

[95] Hori, T., Ikuta, S., Hattori, S., Takao, K., Miyakawa, T. and Koike, C. (2021) Mice with mutations in *Trpm1*, a gene in the locus of 15q13.3 microdeletion syndrome, display pronounced hyperactivity and decreased anxiety-like behavior. Mol. Brain 14, 61 10.1186/s13041-021-00749-y 33785025 PMC8008678

[96] Olsen, L., Sparsø, T., Weinsheimer, S.M., Dos Santos, M.B.Q., Mazin, W., Rosengren, A. et al. (2018) Prevalence of rearrangements in the 22q11.2 region and population-based risk of neuropsychiatric and developmental disorders in a Danish population: a case-cohort study. Lancet Psychiatry 5, 573–580 10.1016/S2215-0366(18)30168-8 29886042 PMC6560180

[97] Papaioannou, V.E. (2014) The T-box gene family: emerging roles in development, stem cells and cancer. Development 141, 3819–3833 10.1242/dev.104471 25294936 PMC4197708

[98] Hiramoto, T., Sumiyoshi, A., Yamauchi, T., Tanigaki, K., Qian, S., Kang, G. et al. (2022) *Tbx1*, a gene encoded in 22q11.2 copy number variant, is a link between alterations in fimbria myelination and cognitive speed in mice. Mol. Psychiatry 27, 929–938 10.1038/s41380-021-01318-4 34737458 PMC9054676

[99] Burger, K. and Gullerova, M. (2015) Swiss army knives: non-canonical functions of nuclear Drosha and Dicer. Nat. Rev. Mol. Cell Biol. 16, 417–430 10.1038/nrm3994 26016561

[100] Amin, H., Marinaro, F., De Pietri Tonelli, D. and Berdondini, L. (2017) Developmental excitatory-to-inhibitory GABA-polarity switch is disrupted in 22q11.2 deletion syndrome: a potential target for clinical therapeutics. Sci. Rep. 7, 15752 10.1038/s41598-017-15793-9 29146941 PMC5691208

[101] Paterlini, M., Zakharenko, S., Lai, W.S., Qin, J., Zhang, H., Mukai, J. et al. (2005) Transcriptional and behavioral interaction between 22q11.2 orthologs modulates schizophrenia-related phenotypes in mice. Nat. Neurosci. 8, 1586–1594 10.1038/221562 16234811

[102] Yao, Y., Jin, C., Liao, Y., Huang, X., Wei, Z., Zhang, Y. et al. (2024) Schizophrenia-like behaviors arising from dysregulated proline metabolism are associated with altered neuronal morphology and function in mice with hippocampal PRODH deficiency. Aging Dis. 15, 1952–1968 10.14336/AD.2023.0902 37815900 PMC11272211

